# End growth results of exercise treatment to avoid bracing in adolescents with idiopathic scoliosis: a prospective cohort controlled study

**DOI:** 10.1186/1748-7161-9-S1-O71

**Published:** 2014-12-04

**Authors:** Stefano Negrini, Sabrina Donzelli, Alessandra Negrini, Silvana Parzini, Michele Romano, Fabio Zaina

**Affiliations:** 1 University of Brescia - IRCCS Don Gnocchi, Brescia, Italy; 2 ISICO - Italian Scientific Spine Institute, Milan, Italy

## Background

Doubts on the efficacy of exercise treatment for adolescents with Idiopathic Scoliosis (IS) still exists.

## Aim

To verify the effectiveness of exercises in everyday clinics.

## Design

Prospective observational controlled cohort study nested in a prospective database started in March 2003.

## Methods

Setting: outpatient tertiary referral clinics.

Participants: consecutive patients from start of the database to 31/12/2010. Inclusion criteria: IS; Risser 0-2; 11° to 20° Cobb; age 10 years or more; first evaluation. Exclusion criteria: consultations only; immediate prescription of a brace.

Groups: Physiotherapic Specific Scoliosis Exercises - SEAS school (PSSE: at least 45 min/week, 3 cognitive-behavioral sessions/year); Controls (CON: less than 15 min/week); Usual Physiotherapy (UP: other institutes/protocols).

End-Of-Treatment (EOT): medical prescription, bracing, Risser 3.

Failures: bracing for scoliosis; EOT above 30°.

Statistical analysis: intent-to-treat (ITT: drop-outs included as failures) and efficacy (EA: only EOT patients). Relative Risk of failure (RR), 95% Confidence Interval (CI), and clinical and radiographic changes have been calculated.

## Results

Out of 327 patients, 34 (10%) were excluded due to bracing at first evaluation. We included 293 adolescents: 145 PSSE, 95 UP, 53 CON, with no differences at baseline. Physicians prescribed bracing (failure) without differences among groups.

Failures and drop-outs were 84 (28.7%) and 47 (16.0%) respectively: 21.4% and 18.6% in PSSE; 33.7% and 9.5% in UP; 39.6% and 20.8% in CON.

Efficacy analysis (RR): CON vs PSSE 1.90 (IC 1.48-2.33); UP vs PSSE 1.42 (1.01-1.82); CON vs UP: not significant.

Intent-to-treat (RR): CON vs PSSE 1.51 (1.21-1.80); CON vs UP 1.40 (1.08-1.72); UP vs PSSE: not significant.

At the end of exercises, aesthetics (TRACE) improved statistically in PSSE (1.8 points out of 12) and UP (1.5), not in CON; only PSSE improvement was statistically better than CON.

## Conclusion

Patients performing UP or nothing (CON), compared to those treated with PSSE (SEAS), increase the risk of failure (bracing and/or 30° at EOT) 1.9 and 1.4 times respectively (EA).

